# Supplementary material for: The Effects of Prophylactic Laxative Use on Critically Ill Patients Requiring Mechanical Ventilation: A Retrospective Cohort Study
Source: Diseases. 2024 Nov 1;12(11):274. doi: 10.3390/diseases12110274 (PMC11593228; doi:10.3390/diseases12110274)

Supplemental Table S1. Diseases and ICD codes

Disease	ICD-9 and ICD-10 codes
Congestive heart failure	'39891','40201','40291','40491','40413','40493', '4280', '4281', '42820', '42821', '42822', '42823', '42830', '42831', '42832', '42833', '42840', '42841','42842', '42843', '4289', '428', '4282', '4283', '4284', 'I501','I509','I502%', 'I503%', 'I508%', 'I504%'
Liver disease	'571%','K7400','K7401','K7402','K741','K742', 'K743','K744','K745','K7460','K7469'
Renal disease	'585%', 'N181','N182','N1830','N1831','N1832','N184','N185','N186','N189'
COPD	'490', '4910', '4911', '49120','49121', '4918', '4919', '4920', '4928','494', '4940', '4941', '496', 'J440', 'J441','J449','J430', 'J431','J439','J432','J438', 'J470','J471', 'J479'
Diabetes	'2535','3572','5881','64800','64801','64802','64803','64804','7751','V771','V180',' V1221'

Abbreviation: COPD, Chronic obstructive pulmonary disease

Supplemental Table S2. The proportion of missing values of all variables

Variable	miss	Percentage (%)
Lactic acid	229	8.927875244
Temperature	202	7.875243665
pO2	93	3.625730994
pCO2	93	3.625730994
pH	85	3.313840156
Weight	47	1.832358674
Chloride	29	1.130604288
HCO3	18	0.701754386
BUN	18	0.701754386
WBC	12	0.467836257
Hb	12	0.467836257
PLT	12	0.467836257
Potassium	8	0.311890838
Sodium	8	0.311890838
SCr	7	0.272904483
CHF	1	0.038986355
Renal	1	0.038986355
Liver	1	0.038986355
COPD	1	0.038986355
Magnesium	1	0.038986355
Laxative	0	0
VFD	0	0
Service Unit	0	0
Age	0	0
Gender	0	0
Death	0	0
APSIII	0	0
Sofa score	0	0
CCI	0	0
DM	0	0
Vasopressor	0	0
ICU-free day	0	0
HR	0	0
MAP	0	0
Enteral nutrition	0	0
C. difficile infection	0	0
Enterobacteria infection	0	0
Hypernatremia	0	0
Hypomagnesemia	0	0

Hypokalemia	0	0
Diarrhea	0	0
VAP	0	0
Laxative	0	0

Abbreviation: BUN, Blood Urea Nitrogen; APSIII, Acute Physiology Score III; SOFA, Sequential Organ Failure Assessment; CCI, Charlson Comorbidity Index; DM, diabetes, CHF, Congestive heart failure; COPD, Chronic obstructive pulmonary disease; HR, heart rate; MAP, Mean Arterial Pressure, WBC, white blood cell; Hb, Hemoglobin; SCr, Serum Creatinine, MICU, medical intensive care unit; SICU, surgical intensive care unit; TSICU, trauma surgical intensive care unit; CCU, coronary care unit; CSRU, cardiac surgery recovery unit.

Supplemental Table S3. Univariate analysis of the association between variables and in-hospital mortality

Variables	OR (95%CI)	P-value
Laxative		
Non-Laxative	Ref	
Stimulant	0.75 (0.55-1.02)	0.07
Docusate	0.54 (0.38-0.75)	<0.001
Combination	1.1 (0.85-1.41)	0.47
Service unit, n (%)		
MICU	Ref	
SICU/TSICU	0.92 (0.75-1.15)	0.47
CCU/CSRU	0.87 (0.68-1.11)	0.26
Age (years)	1.02 (1.02-1.03)	<0.001
Female (%)	1.22 (1.01-1.47)	0.04
Weight (kg)	0.99 (0.99-0.99)	<0.001
APSIII	1.02 (1.02-1.02)	<0.001
SOFA score	1.02 (1.02-1.03)	<0.001
CCI score	1.13 (1.1-1.17)	<0.001
DM (Yes, %)	0.98 (0.8-1.2)	0.85
CHF (Yes, %)	0.94 (0.76-1.15)	0.53
Renal disease (Yes, %)	1.3 (1.02-1.65)	0.03
Liver disease (Yes, %)	1.11 (0.73-1.67)	0.64
COPD (Yes, %)	1.04 (0.81-1.32)	0.78
Vasopressor (Yes, %)	1.44 (1.13-1.82)	<0.001
Temperature (C)	0.83 (0.78-0.88)	<0.001
HR (bmp)	1 (1-1)	0.41
MAP (mmHg)	1 (1-1)	0.06
WBC (K/uL)	1.01 (1-1.02)	0.21
Hb (g/L)	1.01 (0.97-1.05)	0.6
Platelet (K/uL)	1 (1-1)	0.15
pH	0.42 (0.2-0.86)	0.02
pO2 (mmHg)	1 (0.99-1)	<0.001
pCO2 (mmHg)	1 (0.99-1)	0.22
Potassium (mmol/L)	0.99 (0.91-1.08)	0.83
Sodium (mmol/L)	1.02 (1.01-1.04)	0.01
Magnesium (mg/dL)	1.19 (0.92-1.54)	0.19
Chloride (mmol/L)	1 (0.99-1.01)	0.97
BUN (mg/dL)	1.01 (1-1.01)	<0.001
Creatinine (mg/dL)	1.07 (1.01-1.12)	0.01
Lactic acid (mmol/L)	1.07 (1.04-1.1)	<0.001
Enteral nutrition (Yes, %)	0.31	0.31

Abbreviation: BUN, Blood Urea Nitrogen; APSIII, Acute Physiology Score III; SOFA, Sequential Organ Failure Assessment; CCI, Charlson Comorbidity Index; DM, diabetes, CHF, Congestive heart failure; COPD, Chronic

obstructive pulmonary disease; HR, heart rate; MAP, Mean Arterial Pressure, WBC, white blood cell; Hb, Hemoglobin; SCr, Serum Creatinine, MICU, medical intensive care unit; SICU, surgical intensive care unit; TSICU, trauma surgical intensive care unit; CCU, coronary care unit; CSRU, cardiac surgery recovery unit.

Supplemental Table S4. Analysis of the association among laxatives and clinical outcomes with multivariable logistic regression and generalized linear regression

Outcomes	Non-Laxative	Stimulants		Docusate		Stimulants-docusate	
		OR (95% CI)	*P*	OR (95% CI)	*P*	OR (95% CI)	*P*
**Primary outcome**							
In-hospital mortality	Reference	0.86 (0.62 - 1.19)	0.37	0.48 (0.33 - 0.69)	<0.001	1.23 (0.94 - 1.61)	0.13
**Major secondary outcomes**					P		
ICU-free day*	Reference	0.96 (0.89-1.03)	0.211	0.92 (0.85-0.99)	0.028	0.96 (0.901-1.012)	0.167
Ventilator-free day*	Reference	0.98 (0.95-1.02)	0.322	1.01 (0.98-1.05)	0.414	0.99 (0.96-1.02)	0.574
**Other secondary outcomes**							
Diarrhea	Reference	1.03 (0.78 - 1.38)	0.813	0.8 (0.58 - 1.1)	0.163	1.01 (0.79 - 1.3)	0.921
VAP	Reference	0.95 (0.72 - 1.25)	0.712	0.72 (0.53 - 0.97)	0.03	0.88 (0.7 - 1.12)	0.311
Enterobacteria, any	Reference	1.08 (0.77 - 1.52)	0.651	0.92 (0.64 - 1.34)	0.681	0.9 (0.66 - 1.23)	0.519
C. difficile	Reference	0.85 (0.38 - 1.94)	0.706	0.71 (0.29 - 1.74)	0.452	0.88 (0.43 - 1.78)	0.714
Hypernatremia	Reference			1.1 (0.39 - 3.12)	0.859	0.85 (0.31 - 2.32)	0.746
Hypokalemia	Reference	0.75 (0.53 - 1.07)	0.111	1 (0.71 - 1.4)	0.988	0.9 (0.67 - 1.21)	0.479
Hypomagnesemia	Reference	1 (0.71 - 1.42)	0.992	1.08 (0.76 - 1.54)	0.659	0.82 (0.6 - 1.13)	0.232

* Continuous variables use β (95% CI) instead of OR (95% CI).

Abbreviations: IPTW, Inverse probability treatment weighting; OR, odds ratio; CI, confidence interval. VAP: ventilator-associated pneumonia.

Supplemental Figure S1. Results of Schoenfeld residuals of testing proportional hazards assumption

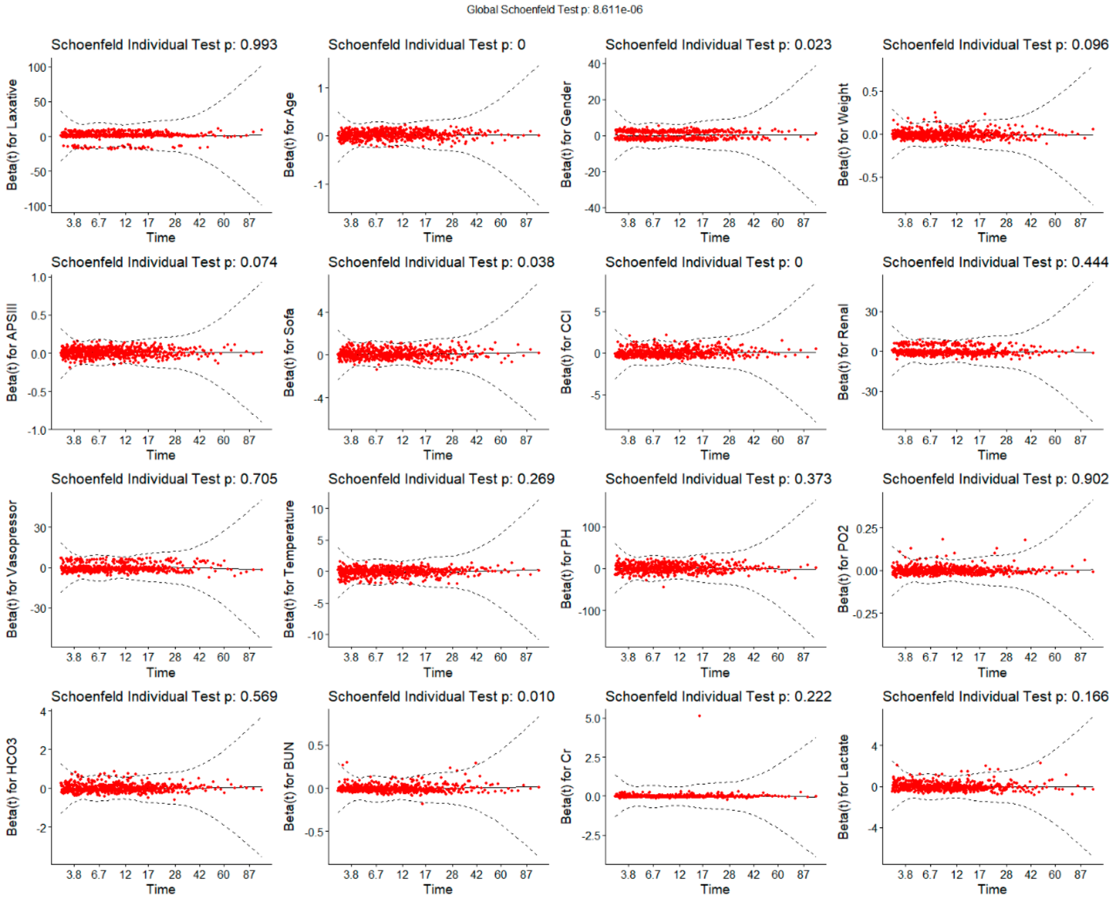

Supplement: Supplementary file 1 [file diseases-12-00274-s001.zip › diseases-3205511-supplementary.pdf]
